# No Profession Exempt

**Published:** 1879-01

**Authors:** Ausburn Towner


### NO PROFESSION EXEMPT.

BY AUSBURN TOWNER.

The following paragraph from the Philadelphia *Transcript* is obtaining a very extensive currency throughout the country:

"Our advice to any one who wants to become an editor is, 'Don't.' Although honorable, journalism is far from gainful. It involves steady, persistent work; a constant strain of the mental faculties, and no end of unintelligent, yet annoying criticism. There is no profession that exacts so much work for so small a return; none in which the steps of promotion are more numerous or more difficult to ascend. The chances of the candidate for journalistic honors for promotion depend upon his industry, his ability, and accident. The demand for service such as he can render is necessarily limited, the supply large and increasing, and he must be content to accept the best position and the best pay that he can get. One thing more must be said, and should be carefully considered by the would-be journalist at the start. Hard as it is to get into journalism, it is harder still to get out. The training which a newspaper affords is not calculated to fit a man for any other known vocation or profession. In nineteen cases out of twenty the novice enters upon it 'for better or for worse,' and for all time. If he can make an honest living in any other way he had better eschew journalism. If he must engage in it, let it be with his eyes open to the difficulties and disappointments that will inevitably beset his path."

The person who wrote the paragraph is either an unsuccessful journalist or has no love for his profession, and went into it for want of something better, as he thinks, to do. Possibly he is both, for no one who is not interested in his work, who does not love it for itself can succeed or expect to succeed. If he had been a clergyman, a physician, a doctor, or a carpenter, his advice would doubtless have been the same, if asked as to the business in which he was engaged. The paragraph can be easily paraphrased and applied to any profession or business and correctly. Try this and every two lawyers in three that you meet will approve it, only they don't have the chance to say it in print:

"Our advice to any one who wants to become a lawyer is, 'Don't.' Although honorable, the legal profession is far from gainful. It involves steady, persistent work; a constant strain of the mental faculties, and no end of unintelligent, yet annoying criticism. There is no profession that exacts so much work for so small a return; none in which the steps of promotion are more numerous or more difficult to ascend. The chances of the candidate for legal honors for promotion depend upon his industry, his ability, and accident. The demand for service such as he can render is necessarily limited, the supply large and increasing, and he must be content to accept the best position and the best pay that he can get. One thing more must be said, and should be carefully considered by the would-be lawyer at the start. Hard as it is to get into law practice, it is harder still to get out. The training which the lawyer's office affords is not calculated to fit a man for any other known vocation or profession. In nineteen cases out of twenty the novice enters upon it 'for better or for worse,' and for all time. If he can make an honest living in any other way he had better eschew the legal profession. If he must engage in it, let it be with his eyes open to the difficulties and disappointments that will inevitably beset his path."

These are the words of disappointed men whatever may be their profession, men who do work in which their hearts are not engaged, and they are true only so far as they apply to themselves.

Success, as it is usually measured, and what one gains in his business, is not the goal of every man's effort. There is a self-satisfaction in doing good work if you don't get a cent for it, and even if some one else gets all the credit besides. There are physicians who take a deep interest in cases; lawyers who give themselves up to their suits; ministers who put their whole souls in their work; and editors, whose best efforts are given to their journals, who, although they reap only a slight, if any pecuniary reward and perhaps attain no prominence, are yet supremely happy in their labors. They bear the disappointments and difficulties, the unintelligent and annoying criticisms that are not inseparable from any calling, with a brave heart, and have compensation in the fact of a self-consciousness that their life is governed by their best and strongest impulses.

In any profession or business every one who follows it cannot be at the top. All clergymen cannot be Wesleys, or Newmans, or Halls; neither can all lawyers be O'Conors, Kents, or Hills; nor all physicians Brown-Sequards, Harveys, or Parkers; nor all editors Greeleys, Bennetts, or Raymonds. The great majority of mankind are those who are never heard of, and it is safer to say to a young man seeking advice as to his future career: To what does your strongest inclination lead you? Whatever that is, follow it. You will not be as happy in a calling that you do not like, even if your income is enormous, as you will, in one to which your whole heart is given, even if your earnings are light. Many a man who has had what the world has called a great success, when he has reached his death-bed, has moaned in spirit over what he esteems a wasted life, wasted, in that he has not done what his heart prompted him to do. And whatever calling you select do not so fervently expect to reach the summit of its excellence that a failure to do so will embitter your life and turn you against your business or profession. Eminence, or a conspicuous position, oftener comes from fortuitous circumstances than from laborious effort. Every one knows men in every profession who are entitled by every consideration to occupy as high places as some who do occupy them, but who have never had an opportunity to show their worth.

An earnest ambition is a good thing to have, but let it be the reasonable one that goes a step upward at a time, and not the one that attempts to reach the top at one single bound, and thus overleaps itself and falls on the other side.

After all, such advice applies only to exceptional cases. The vast majority of young men, although it is certainly true that every one can do one thing better than he can anything else, have no deep craving for any particular profession or business. If they have, you may be sure they will follow it, no matter what difficulties stand in their way, or what obstacles are to be overcome. They have a call for it, and it makes them preach, or teach, or practice medicine, or paint pictures, or write poetry, or build houses, or invent telephones anyhow, in the end, in whatever direction their attention may be at first turned, and in their several walks they are successful.

But that vast majority must do something, and they are the ones who fill up the ranks of all the professions and trades. Having no decided aptitude for any one branch, it makes but little difference which one they select, or is selected for them. They make their business a mere hand-to-mouth, bread-and-butter producer. They go to their duties reluctantly, leave them gladly, and esteem it irksome ever to talk shop except *in* the shop. They see men in other lines and think if they had followed in them, they too would have been successful, and conclude with a belief that they have missed their missions, when it would have made no difference where their lives had been cast, the result would have been the same.

It is fair to presume that the writer of the paragraph that opens this homily also belongs to this vast majority, and feels disgusted with his profession and himself because he is not a Forney, a Childs, or a McMichael. Let him console himself with the reflection that it would have been no different if he had chosen any other profession.

